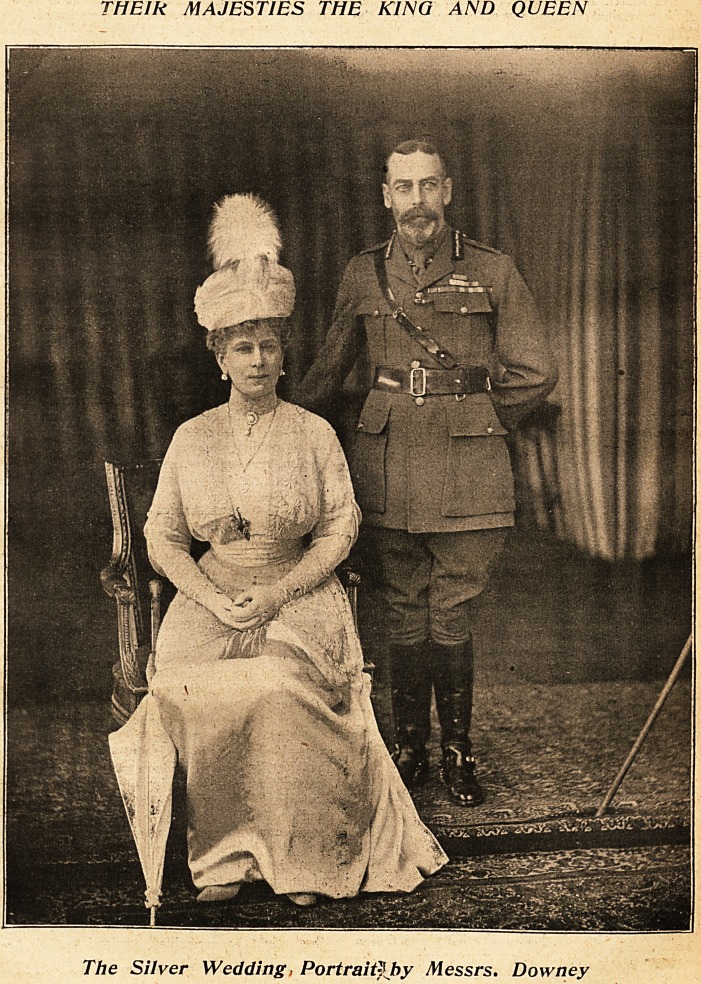# Silver Wedding Portrait of Their Majesties

**Published:** 1918-08-10

**Authors:** 


					August 10, 1918. THE HOSPITAL 411
THElk MAJESTIES THE KING AND QUEEN
The Silver Wedding, Portniity hy Messrs. Downey

				

## Figures and Tables

**Figure f1:**